# Epidemiology and Antimicrobial Resistance Profiles of the *Nocardia* Species in China, 2009 to 2021

**DOI:** 10.1128/spectrum.01560-21

**Published:** 2022-03-02

**Authors:** Hao Wang, Yue Zhu, Qiaozhen Cui, Wenming Wu, Gang Li, Dongke Chen, Lili Xiang, Jiuxin Qu, Dongyan Shi, Binghuai Lu

**Affiliations:** a Department of Clinical Laboratory, Baoding People’s Hospital, Baoding, China; b Laboratory of Clinical Microbiology and Infectious Diseases, Department of Pulmonary and Critical Care Medicine, China-Japan Friendship Hospital, Beijing, China; c Department of Clinical Laboratory, Shanxi Provincial People’s Hospital, Taiyuan, China; d Department of Clinical Laboratory, General Hospital of Ningxia Medical University, Yinchuan, China; e Department of Clinical Laboratory, Beijing Hospital, Beijing, China; f Department of Laboratory Medicine, Chongqing Shapingba District Chenjiaqiao hospital, Affiliated Hospital of Chongqing Medical and Pharmaceutical College, Chongqing, China; g Department of Clinical Laboratory, Shenzhen Third People's Hospital, Southern University of Science and Technology, National Clinical Research Center for Infectious Diseases, Shenzhen, China; h Department of Clinical Laboratory, The Second Hospital of Hebei Medical University, Shijiazhuang, China; University of Texas Southwestern Medical Center

**Keywords:** species distribution, genetic diversity, *Nocardia*, nocardiosis, trimethoprim-sulfamethoxazole

## Abstract

The genus Nocardia includes ubiquitous environmental saprophytes and the most frequently isolated aerobic actinomycete human pathogen responsible for localized or disseminated infection. Herein, the species distribution and antimicrobial susceptibility profiles of 441 nonrepetitive Nocardia strains are reported, collected from 21 provinces/cities in China over 13 years (from 2009 to 2021). These isolates were identified to species level by mass spectrometry or targeted DNA sequencing. The susceptibility profiles of Nocardia species for 15 antibiotics were determined by the broth microdilution method. Among these Nocardia isolates, Nocardia farcinica was the most commonly isolated species (39.9%, 176 of 441), followed by Nocardia cyriacigeorgica (28.6%, 126), Nocardia abscessus (6.6%, 29), and Nocardia otitidiscaviarum (5.9%, 26). Furthermore, 361 Nocardia strains (81.9%) were collected from lower respiratory tract (sputum, lung tissue, and bronchoalveolar lavage fluid), 50 (11.3%) were collected from skin and soft tissues, 9 were collected from blood, 9 were collected from eye, 4 were collected from cerebrospinal fluid and brain abscesses, and 2 were collected from pleural effusion. All of the Nocardia strains were susceptible to linezolid, followed by amikacin (99.3%) and trimethoprim-sulfamethoxazole (TMP-SMX) (99.1%). The antibiotic resistance profiles of other antibiotics varied tremendously among different Nocardia species. This demonstrated that accurate species identification and/or antibiotic susceptibility testing should be performed before the usage of these antibiotics. In summary, this is the largest study on the species and antibiotic resistance profiles of the genus Nocardia circulating in China, and our data will contribute to a better understanding of clinical nocardiosis.

**IMPORTANCE** The genus Nocardia has the potential to cause nocardiosis, which might be underrecognized and underdiagnosed. Herein, the demographical features of 441 nonrepetitive nocardiosis cases and species distribution of their Nocardia strains in China, 2009 to 2021, are summarized. The susceptibility profiles for 15 antibiotics against all of the above Nocardia strains were also determined by the broth microdilution method. To date, this is the largest study on the genus Nocardia contributing to nocardiosis in China. Our study will be helpful for understanding the species diversity of Nocardia isolates distributed in China and for decision-making in the context of nocardiosis diagnosis and treatment.

## INTRODUCTION

The genus Nocardia are Gram-positive, aerobic, and slow-growing actinomycetes universally detected in soil, decaying vegetation, and water (1, 2). Surveillance studies have shown that the organism is responsible for nocardiosis, ranging from localized cutaneous infections to pulmonary and disseminated infections in both immunocompetent and immunosuppressed hosts (1, 3, 4). Nocardiosis constitutes a significant public health care threat due to its underdiagnosis and the lack of sufficient understanding (5).

Ongoing studies have updated the taxonomy of the genus Nocardia and showed that, of 119 recognized Nocardia species with valid names, 54 were related to human infection (6–9), and the number of child taxa of Nocardia species with a validly published and correct name reaches 120 (https://lpsn.dsmz.de/genus/nocardia). The distribution of Nocardia species varies geographically; however, the Nocardia nova complex, Nocardia cyriacigeorgica, Nocardia farcinica, Nocardia brasiliensis, the Nocardia abscessus complex, and the Nocardia transvalensis complex, possibly in a different order, are the six most common species and species complexes identified (10–13). Most Nocardia strains remained susceptible to amikacin, linezolid, and trimethoprim-sulfamethoxazole (TMP-SMX) but have varied susceptibility profiles to β-lactam antibiotics, fluoroquinolones, and others (6, 10–13).

The characteristics of nocardiosis in China are scantly described, except for a few small studies in recent years (2, 14). Nocardiosis is most likely underreported due to problems with laboratory detection. The species distribution and susceptibility profiles of Nocardia isolates show geographic variation and change over time and require continuous surveillance. Herein, we enrolled 441 clinical isolates of Nocardia species circulating in China and reported the species distribution, infection sites, and antibiotic resistance panels. To our knowledge, this is the largest-sample-size surveillance study on Nocardia strains and nocardiosis throughout China.

## RESULTS

### Demographic data of 441 nocardiosis cases.

**FIG 1:**
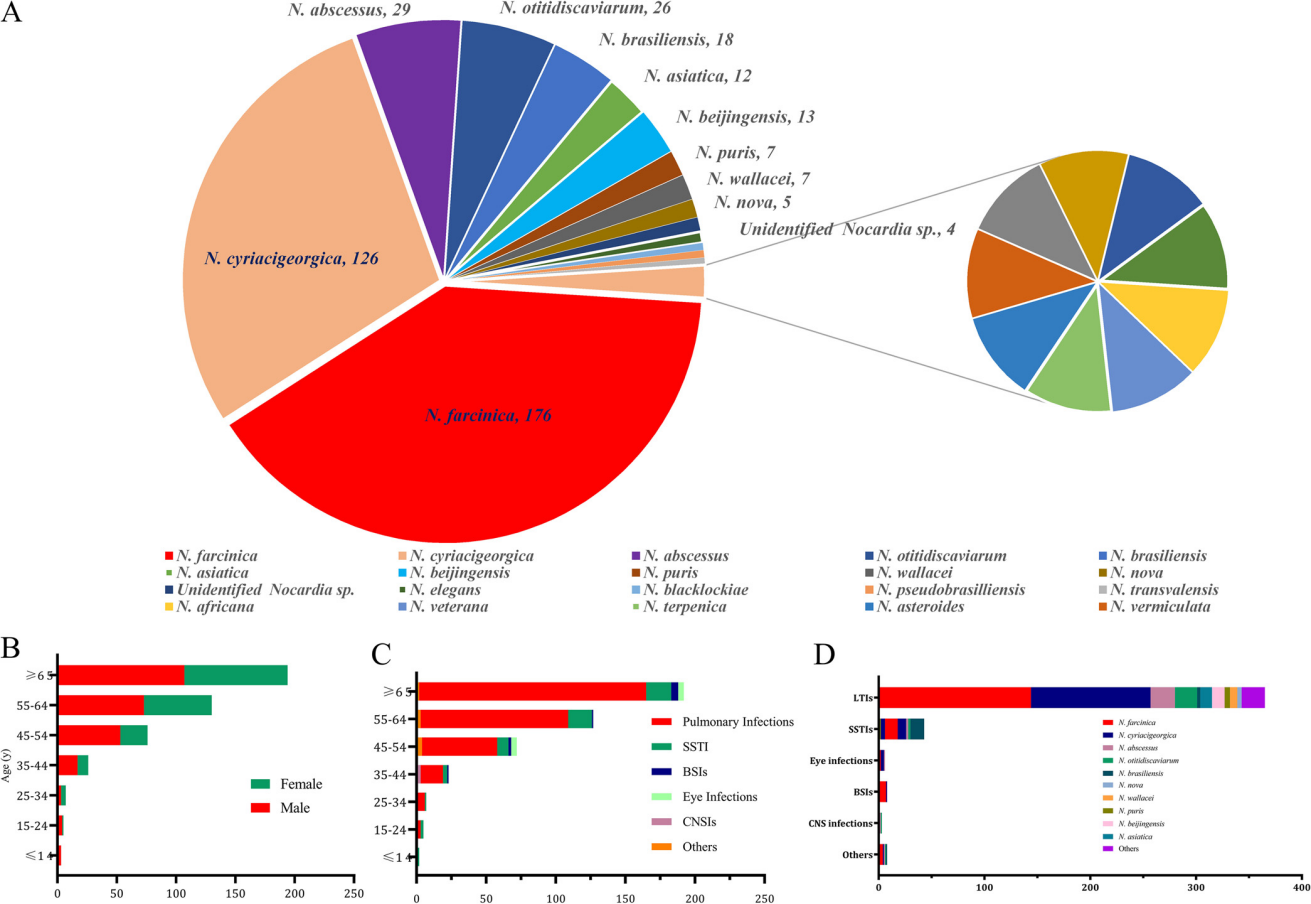
Demographic features of *Nocardia* isolates and nocardiosis patients. (A) Species distribution of 441 *Nocardia* isolates. (B) Correlation between ages and genders of the enrolled nocardiosis patients. (C) Correlation between ages and infection types caused by *Nocardia* spp. (D) Correlation between the commonly isolated *Nocardia* species and infection types. LTI, low respiratory tract infection; SSTI, skin and soft tissue infection; BSI, bloodstream infection; CNSI, central nervous system infection.

The basic characteristics of 441 nocardiosis cases were summarized in Tables 1 and 2. Of 441 Nocardia isolates, 361 (81.9%) were recovered from the lower respiratory tract (from sputum [304], bronchoalverolar lavage fluid [BALF, 54], and lung tissue [3]); 50 (11.3%) were recovered from skin wound, pus, abscess, and soft tissue; 9 were recovered from blood; 9 were recovered from the eye (artificial eye secretion, eyelid abscess, and eye pus/secretion in those with Nocardia keratitis), 4 were recovered from cerebrospinal fluid (CSF) and brain abscesses; 3 were recovered from joint puncture fluid; 2 were recovered from pleural effusion; 1 was recovered from liver abscess; 1 was recovered from pericarditis; and 1 was recovered from ascites (Fig. 1; Table 2).

**TABLE 1:** Demographic and clinical characteristics of 441 nocardiosis patients from 2009 to 2021 in China

Characteristic	No.	Percentage (%)
Patient demographics		
Mean age (range) (ys)	61.6 (6 to 91)	
≤14	3	0.7
15 to 24	5	1.1
25 to 34	7	1.6
35 to 44	26	5.9
45 to 54	76	17.2
55 to 64	130	29.5
≥65	194	44.0
Sex		
Male	260	59.0
Female	181	41.0
Year *Nocardia* isolates collected		
2009	1	0.2
2010	1	0.2
2011	2	0.5
2012	2	0.5
2013	6	1.4
2014	2	0.5
2015	19	4.3
2016	19	4.3
2017	35	7.9
2018	100	22.7
2019	179	40.6
2020	67	15.2
2021	8	1.8
Infection types and sample sources		
Pulmonary nocardiosis		
Sputum	304	68.9
Bronchoalveolar lavage fluid	54	12.2
Lung tissue	3	0.7
Skin and subcutaneous nocardiosis		
Skin and soft tissue pus	50	11.3
** **Central nervous system nocardiosis	
Cerebrospinal fluid	2	0.5
Brain abscess	2	0.5
Laboratory-confirmed bloodstream nocardiosis		
Blood	9	2.0
Others		
Eye pus or excretion	9	2.0
Joint fluid	3	0.7
Pleural effusion	2	0.5
Pericardial effusion	1	0.2
Liver abscess	1	0.2
Ascites	1	0.2

**TABLE 2:** Distribution of age and infection types of 441 nocardiosis cases in China

Infection type	≤14	15 to 24	25 to 34	35 to 44	45 to 54	55 to 64	≥65	Total
Pulmonary infection	1	3	6	19	58	109	165	361
Skin and soft tissue infection	1	2	1	3	8	17	18	50
Bloodstream infection				1	2	1	5	9
Eye infection	1				4		4	9
Central nervous system infection				3		1		4
Bone-joint infection					1	2		3
Pleural effusion					2			2
Peritonitis							1	1
Pericarditis					1			1
Liver abscess							1	1
Total	3	5	7	26	76	130	194	441

The enrolled patients were aged from 6 to 91 years, with the mean age of 61.6 years and 44.0% (194 of 441) of  ≥65 years and only 9.3% (41 of 441) of <45 years, as shown in Fig. 1. The gender ratio of male/female was approximately 1.44:1 (260/181).

### Distribution of Nocardia species.

Among the 441 Nocardia isolates, 23 species were identified, with the clinical features shown in Fig. 1. The mostly isolated Nocardia species contains N. farcinica (39.9%, 176), N. cyriacigeorgica (28.6%, 126), N. abscessus (6.6%, 29), Nocardia otitidiscaviarum (5.9%, 26), N. brasiliensis (4.1%, 18), Nocardia beijingensis (2.9%, 13), and Nocardia asiatica (2.7%, 12), respectively. These 7 Nocardia species accounted for 90.7% (400 of 441) of all collected isolates. Furthermore, the rarely isolated Nocardia spp. contains Nocardia blacklockiae (2 isolates), Nocardia pseudobrasilliensis (2), N. transvalensis (2), Nocardia africana (1), Nocardia veterana (1), Nocardia terpenica (1), N. asteroides (1), Nocardia vermiculata (1), Nocardia concave (1), Nocardia carnea (1), Nocardia niigatensis (1), and Nocardia neocaledoniensis (1).

Four Nocardia strains will be defined as “unidentified.” Their genomic sequencing files have been deposited at https://submit.ncbi.nlm.nih.gov/subs/wgs under GenBank accession numbers SAMN24830555 (NK-065), SAMN24830556 (NK-136), SAMN24830557 (NK-203), and SAMN24830558 (NK-S21).

### Antibiotic susceptibility profiles.

**FIG 2:**
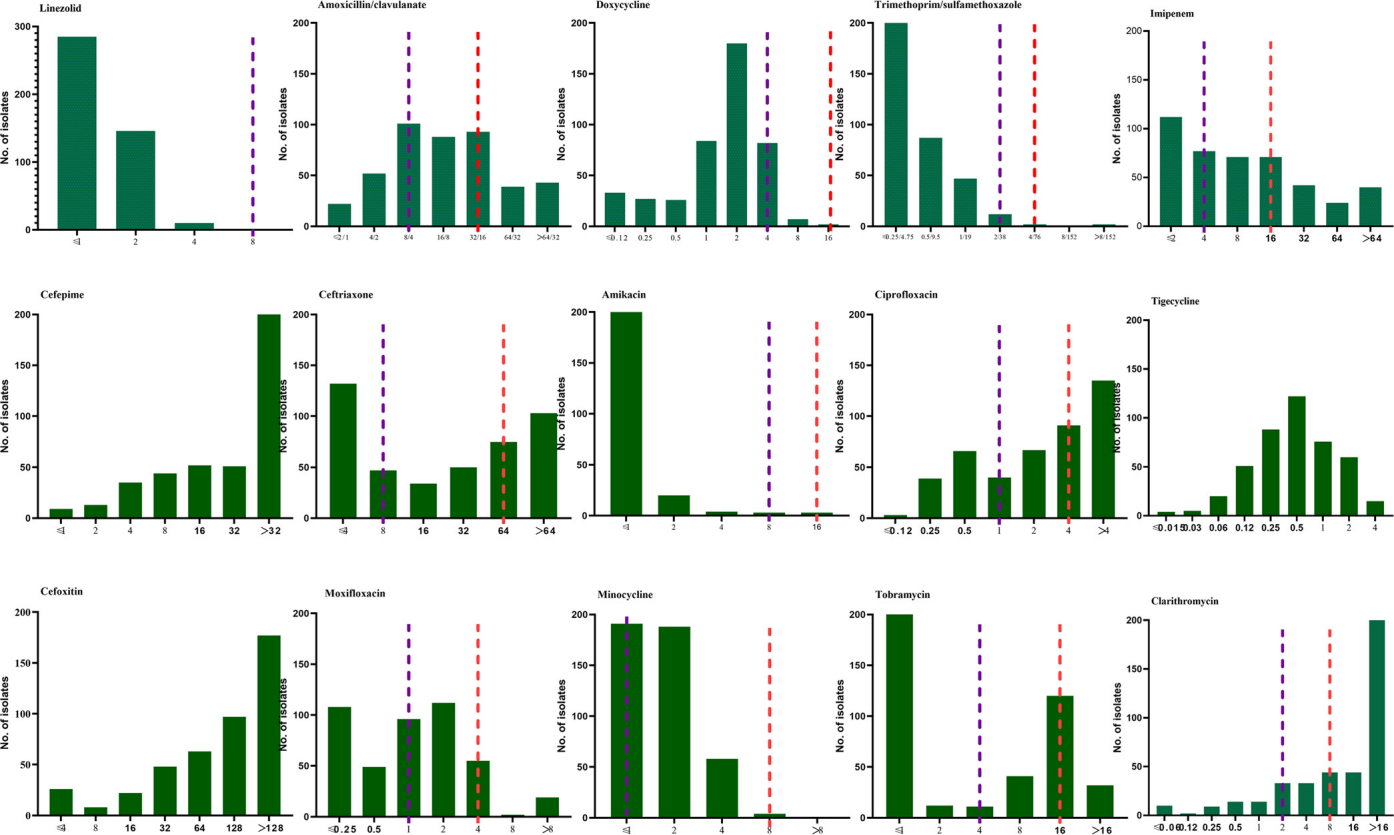
The distribution of the MICs of 441 clinical unique strains of *Nocardia* species for 15 antibiotics. The red and purple dotted lines indicate the resistant and susceptible breakpoints, respectively, for *Nocardia* spp. by Clinical and Laboratory Standards Institute standard M62 (15).

The susceptibility profiles to 15 antibiotics for Nocardia strains are summarized in Table 3 and Fig. 2, showing the MIC_50_, MIC_90_, and resistance rates for each antibiotic. All Nocardia strains were susceptible to linezolid, followed by amikacin (99.3%; 3 of 7 Nocardia wallacei were amikacin-resistant) and TMP-SMX (99.1%; all 4 resistant strains belong to N. farcinica).

**TABLE 3:** Antimicrobial susceptibilities profiles and MIC values to 15 antibiotics of the major Nocardia species/complex in clinical infections in China from 2009 to 2021^*a*^

Drugs	Breakpoint		Species/complex, no. of strains (%)^*b*^										
			*N. farcinica*, 176 (39.9)	*N. cyriacigeorgica*, 126 (28.6)	*N. abscessus* complex, 54 (12.2)^*c*^	*N. otitidiscaviarum*, 26 (5.9)	N. brasiliensis, 18 (4.1)	*N. transvalensis* complex, 11 (2.5)^*d*^	*N. nova* complex, 11 (2.5)^*e*^	*N. puris*, 7 (1.6)	*N. pseudobrasiliensis*, 2 (0.5)	Other *Nocardia*, 10 (2.3)^*f*^	All *Nocardia*, 441 (100)
TMP-SMX	S ≤ 2/38, R ≥ 4/76	MIC_50_	≤0.25/4.75	≤0.25/4.75	≤0.25/4.75	0.5/9.5	≤0.25/4.75	≤0.25/4.75	≤0.25/4.75	≤0.25/4.75		≤0.25/4.75	≤0.25/4.75
		MIC_90_	1/19	0.5/9.5	0.5/9.5	2/38	≤0.25/4.75	0.5/9.5	0.5/9.5	0.5/9.5		1/19	1/19
		Range	≤0.25/4.75 to >8/152	≤0.25/4.75 to 1/19	≤0.25/4.75 to 1/19	≤0.25/4.75 to 2/38	≤0.25/4.75 to 0.5/9.5	≤0.25/4.75 to 1/19	≤0.25/4.75 to 0.5/9.5	≤0.25/4.75 to 0.5/9.5		≤0.25/4.75 to 2/38	≤0.25/4.75 to >8/152
		S/R (%)	97.7/2.3	100/0	100/0	100/0	100/0	100/0	100/0	100/0	100/0	100/0	99.1/0.9
Linezolid	S ≤ 8	MIC_50_	2	≤1	≤1	≤1	≤1	≤1	≤1	≤1		≤1	≤1
		MIC_90_	2	2	≤1	2	2	≤1	2	2		2	2
		Range	≤1 to 4	≤1 to 4	≤1 to 2	≤1 to 2	≤1 to 2	≤1 to 2	≤1 to 2	≤1 to 2		≤1 to 2	≤1 to 4
		S/NS (%)	100/0	100/0	100/0	100/0	100/0	100/0	100/0	100/0	100/0	100/0	100/0
Ciprofloxacin	S ≤ 1, R ≥ 4	MIC_50_	0.5	>4	4	2	4	0.5	>4	4		4	4
		MIC_90_	2	>4	>4	>4	>4	2	>4	>4		>4	>4
		Range	≤0.12 to >4	0.5 to >4	0.5 to >4	0.5 to >4	4 to >4	≤0.12 to >4	2 to >4	4 to >4		0.25 to >4	≤0.12 to >4
		S/I/R (%)	68.8/22.2/9.1	2.4/1.6/96	18.5/16.7/64.8	7.7/42.3/50	0/0/100	72.7/18.2/9.1	0/18.2/81.8	0/0/100	50/0/50	30/10/60	33.6/15/51.5
Imipenem	S ≤ 4, R ≥ 16	MIC_50_	8	4	8	>64	>64	16	≤2	4		16	8
		MIC_90_	32	32	>64	>64	>64	32	4	32		64	64
		Range	≤2 to >64	≤2 to >64	≤2 to >64	4 to >64	16 to >64	≤2 to >64	≤2 to 16	≤2 to 32		≤2 to >64	≤2 to >64
		S/I/R (%)	39.8/19.9/40.3	58.7/19/22.2	46.3/18.5/35.2	3.8/0/96.2	0/0/100	36.4/0/63.6	90.9/0/9.1	57.1/14.3/28.6	0/0/100	30/10/60	43.3/16.1/40.6
Moxifloxacin	S ≤ 1, R ≥ 4	MIC_50_	≤0.25	2	2	2	1	≤0.25	2	2		1	1
		MIC_90_	1	4	>8	4	1	0.5	4	4		4	4
		Range	≤0.25 to >8	≤0.25 to >8	≤0.25 to >8	≤0.25 to 4	0.5 to 2	≤0.25 to 2	1 to 4	1 to 4		≤0.25 to >8	≤0.25 to >8
		S/I/R (%)	90.3/8/1.7	18.3/48.4/33.3	29.6/25.9/44.4	46.2/42.3/11.5	94.4/5.6/0	90.9/9.1/0	27.3/54.5/18.2	42.9/42.9/14.3	100/0/0	70/10/20	57.1/25.4/17.5
Cefepime^*c*^		MIC_50_	>32	16	4	>32	>32	8	8	>32		32	>32
		MIC_90_	>32	>32	32	>32	>32	>32	16	>32		>32	>32
		Range	2 to >32	≤1 to >32	≤1 to >32	2 to >32	8 to >32	4 to >32	≤1 to >32	32 to >32		2 to >32	≤1 to >32
Cefoxitin^*c*^		MIC_50_	128	128	8	>128	128	128	64	32		64	128
		MIC_90_	>128	>128	128	>128	>128	>128	64	64		>128	>128
		Range	≤4 to >128	≤4 to >128	≤4 to >128	128 to >128	64 to >128	32 to >128	≤4 to 128	16 to 64		8 to >128	≤4 to >128
Amoxicillin-clavulanic acid	S ≤ 8/4, R ≥ 32/16	MIC_50_	8/4	32/16	16/8	>64/32	8/4	4/2	64/32	32/16		64/32	16/8
		MIC_90_	32/16	64/32	>64/32	>64/32	8/4	16/8	>64/32	64/32		>64/32	>64/32
		Range	≤2/1 to >64/32	≤2/1 to >64/32	≤2/1 to >64/32	≤2/1 to >64/32	≤2/1 to 16/8	4/2 to 32/16	4/2 to >64/32	16/8 to 64/32		8/4 to >64/32	≤2/1 to >64/32
		S/I/R (%)	61.9/22.7/15.3	6.3/27/66.7	48.1/7.4/44.4	11.5/0/88.5	94.4/5.6/0	63.6/27.3/9.1	18.2/18.2/63.6	0/28.6/71.4	50/50/0	10/10/80	39.5/20/40.6
Amikacin	S ≤ 8, R ≥ 16	MIC_50_	≤1	≤1	≤1	≤1	≤1	≤1	≤1	≤1		≤1	≤1
		MIC_90_	2	≤1	≤1	≤1	≤1	16	≤1	≤1		≤1	≤1
		Range	≤1 to 4	≤1 to 8	≤1 to 2	≤1 to 2	to ≤1	≤1 to 16	∼≤1	∼≤1		≤1 to 8	≤1 to 16
		S/R (%)	100/0	100/0	100/0	100/0	100/0	72.7/27.3	100/0	100/0	100/0	100/0	99.3/0.7
Ceftriaxone	S ≤ 8, R ≥ 64	MIC_50_	64	≤4	≤4	>64	16	≤4	8	32		8	32
		MIC_90_	>64	64	8	>64	>64	64	16	>64		>64	>64
		Range	≤4 to >64	≤4 to >64	≤4 to >64	8 to >64	≤4 to >64	≤4 to 64	≤4 to 32	≤4 to >64		≤4 to >64	≤4 to >64
		S/I/R (%)	8.5/25.6/65.9	66.7/17.5/15.9	90.7/7.4/1.9	3.8/3.8/92.3	27.8/27.8/44.4	63.6/18.2/18.2	72.7/27.3/0	42.9/14.3/42.9	50/50/0	60/10/30	40.6/19.3/40.1
Doxycycline	S ≤ 1, R ≥ 8	MIC_50_	2	2	0.25	1	2	2	4	1		1	2
		MIC_90_	4	2	2	2	4	4	8	2		4	4
		Range	≤0.12 to 16	≤0.12 to 4	≤0.12 to 4	≤0.12 to 4	0.5 to 4	1 to 4	0.25 to 8	≤0.12 to 2		≤0.12 to 8	≤0.12 to 16
		S/I/R (%)	18.8/78.4/2.8	47.6/52.4/0	83.3/16.7/0	53.8/46.2/0	22.2/77.8/0	18.2/81.8/0	27.3/45.5/27.3	57.1/42.9/0	50/50/0	50/40/10	38.8/59.2/2
Minocycline	S ≤ 1, R ≥ 8	MIC_50_	2	2	≤1	≤1	≤1	2	2	≤1		2	2
		MIC_90_	4	2	2	2	2	2	4	2		8	4
		Range	≤1 to 8	≤1 to 4	≤1 to 4	≤1 to 4	≤1 to 2	≤1 to 4	≤1 to 8	≤1 to 2		≤1 to 8	≤1 to 8
		S/I/R (%)	26.1/73.3/0.6	42.9/57.1/0	79.6/20.4/0	61.5/38.5/0	83.3/16.7/0	27.3/72.7/0	36.4/54.5/9.1	85.7/14.3/0	50/50/0	40/40/20	43.5/55.6/0.9
Tigecycline^*c*^		MIC_50_	1	0.5	0.25	0.12	0.12	0.5	0.5	0.25		0.5	0.5
		MIC_90_	2	1	1	1	0.25	2	2	1		1	2
		Range	0.03 to 4	≤0.015 to 4	≤0.015 to 4	≤0.015 to 2	0.03 to 0.5	0.12 to 2	0.06 to 2	0.06 to 1		0.06 to 2	≤0.015 to 4
Tobramycin	S ≤ 4, R ≥ 16	MIC_50_	16	≤1	≤1	2	≤1	>16	8	≤1		≤1	≤1
		MIC_90_	>16	≤1	8	16	≤1	>16	>16	≤1		8	16
		Range	≤1 to >16	≤1 to 16	≤1 to >16	≤1 to >16	∼≤1	4 to >16	≤1 to >16	∼≤1		≤1 to 16	≤1 to >16
		S/I/R (%)	14.2/13.6/72.2	96/0.8/3.2	87/7.4/5.6	57.7/23.1/19.2	100/0/0	9.1/18.2/72.7	45.5/27.3/27.3	100/0/0	100/0/0	80/10/10	56.5/9.3/34.2
Clarithromycin	S ≤ 2, R ≥ 8	MIC_50_	>16	16	2	>16	8	8	0.25	>16		1	>16
		MIC_90_	>16	>16	>16	>16	>16	>16	>16	>16		>16	>16
		Range	0.5 to >16	0.12 to >16	≤0.06 to >16	≤0.06 to >16	2 to >16	0.25 to >16	≤0.06 to >16	∼>16		≤0.06 to >16	≤0.06 to >16
		S/I/R (%)	4/1.7/94.3	19.8/15.9/64.3	53.7/9.3/37	3.8/0/96.2	11.1/16.7/72.2	27.3/9.1/63.6	72.7/0/27.3	0/0/100	50/50/0	60/0/40	18.6/7.5/73.9

a The table shows the antimicrobial susceptibilities profiles and MIC values (in μg/mL) (as determined by the broth microdilution method) to 15 antibiotics of the major Nocardia species/complex responsible for clinical infections in China from 2009 to 2021. TMP-SMX, trimethoprim-sulfamethoxazole. S, susceptible; I, intermediate; R, resistant; NS, nonsusceptible; MIC_50_ and MIC_90_, MICs at which 50% and 90% of the strains were inhibited, respectively.

b Percentage with respect to the total number of identified Nocardia strains (*n* = 441).

c N. abscessus complex (54) includes N. abscessus (29), N. asiatica (12), and N. beijingensis (13).

d N. transvalensis complex (11) includes N. wallacei (7), N. blacklockiae (2), and N. transvalensis (2).

e N. nova complex (11) includes N. africana (1), N. elegans (3), N. nova (5), N. vermiculata (1), and N. veterana (1).

f Other Nocardia species (10) includes N. terpenica (1), N. niigatensis (1), N. carnea (1), N. asteroides (1), N. concava (1), N. neocaledoniensis (1), and unidentified Nocardia (4).

For tetracyclines, doxycycline and minocycline-resistant Nocardia accounted for 2.0 and 0.9%, respectively, but the intermediate rates were high: 59.2% and 55.6%, respectively. Tigecycline showed low MIC values against different Nocardia species, with its MIC_90_ at 2 μg/mL. However, for macrolides, 73.9% Nocardia strains were resistant to clarithromycin. For β-lactam antibiotics, including imipenem, cefepime, cefoxitin, amoxicillin-clavulanic acid, and ceftriaxone, all demonstrated a poor performance against Nocardia spp. and high heterogeneity between Nocardia species, as shown in Table 3, suggesting the critical role of AST before the usage of these antibiotics.

The antibiotic resistance profiles varied within different Nocardia species. The resistance rates of N. farcinica to clarithromycin (94.3%), ceftriaxone (65.9%), and imipenem (40.3%) were relatively high, but N. farcinica showed a low resistance rate to fluoroquinolones ciprofloxacin (9.1%) and moxifloxacin (1.7%). The resistance rates of N. cyriacigeorgica to ciprofloxacin and clarithromycin were 96.0 and 64.3%; by comparison, its rates of resistance to doxycycline and minocycline were 0. For the N. abscessus complex and N. nova complex, ceftriaxone was the β-lactam antibiotic most frequently taken as being active (only 1.9 and 0% of isolates were resistant, respectively); however, 35.2 and 9.1% of them were resistant to imipenem.

### Correlation of antimicrobial susceptibility profiles with Nocardia species designation.

The antimicrobial susceptibility pattern types in the present study were compared with those provided by CLSI standard M62 (15). Furthermore, the antimicrobial susceptibility patterns of the well-recognized species are listed for comparison in Table 4. A strong correlation between the drug pattern types and Nocardia species identification was identified and demonstrated. However, some discrepancies were noted; e.g., the N. farcinica isolates, 68.8 and 61.9% of which were susceptible to ciprofloxacin and amoxicillin-clavulanic acid, whose drug patterns were determined to be susceptible by CLSI standard M62.

**TABLE 4:** Correlation and comparison of antimicrobial susceptibility profiles with Nocardia species or complexes designation^*a*^

Similar studies	Involved *Nocardia* species/complexes	No. of *Nocardia* isolates	Susceptibility (%)^*b*^											
			Linezolid	Amikacin	TMP-SMX	Ciprofloxacin	Moxifloxacin	Ceftriaxone	Imipenem	Amoxicillin-clavulanic acid	Doxycycline	Minocycline	Tobramycin	Clarithromycin
*N. farcinica*														
Expected pattern^*c*^	*N. farcinica*		S	S	S	S	NA	R	V	S	NA	V	R	R
This study	*N. farcinica*	176	100	100	97.7	68.8	90.3	8.5	39.8	61.9	18.8	26.1	14.2	4.0
Study 1 (6)	*N. farcinica*, *N. kroppenstedtii*	319	100	100	99	49	76	3	83	96	2	7	1	0
Study 2 (11)	*N. farcinica*	38	100	100	73.7	28.9	50	2.6	5.3	23.7	2.6	2.6	2.6	2.6
Study 3 (12)	*N. farcinica*	204	100	100	99.5	43	79	3	33	76	ND	5	0.5	0.5
Study 4 (13)	*N. farcinica*	36	100	100	94	50	81	6	53	78	17	36	0	0
*N. cyriacigeorgica*														
Expected pattern^*c*^	*N. cyriacigeorgica*		S	S	S	R	NA	S	S	R	NA	V	S	R
This study	*N. cyriacigeorgica*	126	100	100	100	2.4	18.3	66.7	58.7	6.3	47.6	42.9	96	19.8
Study 1 (6)	*N. cyriacigeorgica*	352	100	99	100	0	1	64	99	8	11	14	99	1
Study 2 (11)	*N. cyriacigeorgica*	61	100	100	100	0	0	80.3	6.6	3.3	16.4	8.2	100	0
Study 3 (12)	*N. cyriacigeorgica*	264	100	100	100	0	4	88	43	3	ND	6	99.2	1
Study 4 (13)	*N. cyriacigeorgica*	20	100	100	100	0	0	95	90	15	50	85	100	25
*N. abscessus*														
Expected pattern^*c*^	*N. abscessus* complex		S	S	S	R	NA	S	V	S	NA	V	V	R
This study	*N. abscessus*, *N. asiatica*, *N. beijingensis*	54	100	100	100	18.5	29.6	90.7	46.3	48.1	83.3	79.6	87	53.7
Study 1 (6)	*N. abscessus*, *N. arthritidis*, *N. asiatica*, *N. beijingensis*, *N. pneumoniae*	205	100	100	100	3	13	93	64	61	87	94	100	38
Study 2 (11)	ND	4	100	100	100	0	0	75	0	50	100	75	100	50
Study 3 (12)	*N. abscessus*, *N. arthritidis*, *N. asiatica*	110	100	100	100	0	8	98	31	78	ND	85	100	29
Study 4 (13)	*N. abscessus*, *N. abscessus/arthritidis*-like species cluster	9	100	100	100	11	11	100	22	100	89	89	100	11
*N. otitidiscaviarum*														
Expected pattern^*c*^	*N. otitidiscaviarum*		S	S	S	S	NA	R	R	R	NA	V	V	V
This study	*N. otitidiscaviarum*	26	100	100	100	7.7	46.2	3.8	3.8	11.5	53.8	61.5	57.7	3.8
Study 1 (6)	*N. otitidiscaviarum*	30	100	100	87	0	23	0	3	0	43	60	53	17
Study 2 (11)	*N. otitidiscaviarum*	2	ND	ND	ND	ND	ND	ND	ND	ND	ND	ND	ND	ND
Study 3 (12)	*N. otitidiscaviarum*	29	100	100	100	7	35	0	7	0	ND	45	62	7
Study 4 (13)	*N. otitidiscaviarum*	6	100	100	83	0	17	0	0	0	17	33	50	0
N. brasiliensis														
Expected pattern^*c*^	N. brasiliensis		S	S	S	R		V	R	S		S	S	R
This study	N. brasiliensis	18	100	100	100	0	94.4	27.8	0	94.4	22.2	83.3	100	11.1
Study 1 (6)	N. brasiliensis	223	100	100	100	0	40	2	8	99	5	16	100	0
Study 2 (11)	N. brasiliensis	52	100	100	98.1	0	78.8	38.5	0	90.4	11.5	15.4	100	0
Study 3 (12)	N. brasiliensis	148	100	100	100	1	99	49	1	95.3	ND	24	100	3
Study 4 (13)	N. brasiliensis	6	100	100	100	0	67	33	17	100	17	67	100	0
*N. transvalensis*														
Expected pattern^*c*^	*N. transvalensis* complex		S	R	S	S	NA	S	V	V	NA	V	R	R
This study	*N. blacklockiae*, *N. transvalensis*, *N. wallacei*	11	100	72.7	100	72.7	90.9	63.6	36.4	63.6	18.2	27.3	9.1	27.3
Study 1 (6)	*N. blacklockiae*, *N. transvalensis*, *N. wallacei*	121	100	26	88	49	72	64	9	89	10	31	0	2
Study 2 (11)	ND	4	100	50	75	100	100	100	25	75	25	25	0	50
Study 3 (12)	*N. transvalensis*, *N. wallacei*	83	100	28	81	84	100	63	6	47	ND	15	4	4
Study 4 (13)	*N. transvalensis*, *N. wallacei*	5	100	20	80	100	80	100	0	40	20	20	0	20
*N. nova*														
Expected pattern^*c*^	*N. nova* complex		S	S	S	R	NA	S	S	R	NA	V	R	S
This study	*N. nova*, *N. africana*, *N. elegans*, *N. veterana*	11	100	100	100	0	27.3	72.7	90.9	18.2	27.3	36.4	45.5	72.7
Study 1 (6)	*N. nova*, *N. africana*, *N. elegans*, *N. veterana*, *N. aobensis*, *N. cerradoensis*, *N. kruczakiae*, *N. mikamii*, *N. vermiculata*	452	100	100	100	1	3	14	100	4	1	19	3	97
Study 2 (11)	*N. nova*, *N. veterana*	80	100	100	93.8	3.8	6.3	60	96.3	6.3	3.8	15	13.8	95
Study 3 (12)	*N. nova*, *N. africana*, *N. elegans*, *N. veterana*, *N. kruczakiae*, *N. ikamii*	320	100	100	100	1	2	47	99	9	ND	12	13	97
Study 4 (13)	*N. nova*, *N. nova/cerradoensis/kruczakiae/aobensis*-like species cluster	28	100	100	100	0	7	85	93	26	11	56	7	100
All *Nocardia* species														
This study	All *Nocardia* species	441	100	99.3	99.1	33.6	57.1	40.6	43.3	39.5	38.8	43.5	56.5	18.6
Study 1 (6)	All *Nocardia* species	2091	100	94.1	98	15.8	30.3	36	73.2	44.3	18.7	29.7	51.8	34.8
Study 2 (11)	All *Nocardia* species	270	100	99.3	90.7	11.5	32.2	53	33.7	25.2	12.6	14.4	58.1	37.4
Study 3 (12)	All *Nocardia* species	1299	100	95	98	17	40	56	49	37	ND	22	55	33
Study 4 (13)	All *Nocardia* species	149	100	99	97	22	40	65	59	40	37	61	53	37

a For study 1 (6), the MICs reported as intermediate (I) were combined with resistant (R) for this study. NA, the expected antimicrobial susceptibility patterns are not available; ND, not determined.

b The numbers in this section represent percentages of susceptibility (%) of the corresponding *Nocardia* species or species complexes.

c Expected antimicrobial susceptibility patterns of the most commonly isolated *Nocardia* species or species complexes provided by CLSI standard M62 (15); the expected pattern “R/S/V” represents resistant/susceptible/variable pattern of the *Nocardia* species or species complexes, respectively.

## DISCUSSION

The genus Nocardia are the most commonly isolated aerobic actinomycete genera from clinically significant specimens. There is a paucity of laboratory and clinical data from studies with the distribution and antibiotic resistance profiles of Nocardia species circulating in China (2, 14).

The incidence of nocardiosis is age specific, with the maximum rates observed in elderly patients. In line with our data, the nocardiosis patients had an average age of 61.6 years; 44.0% were ≥65 years old and 9.3% were <45 years, similar to previous documentation (6). The gender ratio of male/female was approximately 1.44:1, in line with what has been previously described in the literature: 1.28 (1,175/916) and 1.13 (1,165/1,033) in two U.S. studies (6, 12) and 1.38 (432/311) in a French study (16). That there is a slight preponderance of males might be partially explained by the fact that males are more likely to come into contact with contaminated soil while working outdoors. Further underlying mechanisms should be explored.

Human nocardiosis might cover localized cutaneous infections from direct inoculation of the skin or soft tissues in immunocompetent people and pulmonary infections by inhalation and disseminated infections in immunosuppressed hosts (1, 17–19). In our study, the specimens from the respiratory tract and from the skin and soft tissues account for large percentages (81.9 and 11.3%, respectively). Nocardia bacteremia is a rare and underreported disease (20), accounting for 2.0% (9 of 441) in our study, 2.2% (6 of 270) in an Australian study (11), and 5.2% (110 of 2,091) in a U.S. study (6). Of the total 9 cases with bacteremia, the age range was 37 to 75 years (including 5 patients who were ≥65 years old), and 4 were male.

The genus Nocardia has had a conflicted and confusing taxonomic history (7). More than 50 species of Nocardia have been identified to be implicated as the cause of serious human infections, and the various Nocardia species have different geographic prevalence (17, 21). In the present study, 23 Nocardia species were identified, of which the 7 most-isolated Nocardia species and species complexes constituted approximately 90%. N. farcinica, N. cyriacigeorgica, and N. abscessus were the most frequently isolated. The distribution of species in our study was different from that reported in recent studies from Spain (1,119 isolates) (4) and the United States (1,299 isolates) (12), where N. cyriacigeorgica and the N. nova complex were the most-identified species. Our study was similar to the results from a French study in which N. farcinica was the most frequently isolated species, accounting for 20.2% (160 of 793) (16). Other species appear with less frequency in clinical settings. No clinical isolates of Nocardia brevicatena/paucivorans (type II drug pattern) were isolated, similar to the results of previous studies (6, 11–13).

The Nocardia species have closely correlated with infection sites, as already reported, N. farcinica was more likely isolated in blood cultures and brain abscesses/cerebrospinal fluid: 21 of 39 (54%) and 19 of 43 (44.2%), respectively (16). In a systematic review of 138 cases of Nocardia bacteremia, 83 were identified to species level, and 55.4% (46 of 83) were N. farcinica (20). In our study, in 9 bacteremia cases, 7 were N. farcinica. Moreover, N. brasiliensis was related predominately to skin and soft tissue infections (1). In our study, of 18 N. brasiliensis isolates, only 3 were collected from respiratory infections, while 13, 1, and 1 were recovered from the cutaneous tissue infections, eye infections, and bone and joint infections, respectively.

Accurate species-level identification is therefore especially important for nocardiosis due to the discrepancies of drug patterns associated with several clinically significant species. The antimicrobial susceptibility profile was highly variable between the Nocardia species, but in general, only amikacin, linezolid, and TMP-SMX demonstrated good *in vitro* activity against most species, as already reported (6, 16). Currently, TMP-SMX constitutes the mainstay of antimicrobial therapy for nocardiosis. The resistance rates of TMP-SMX to Nocardia varied greatly among different geographic areas and the AST methods used. For example, the resistance rate of TMP-SMX was 16.2% (181 of 1,119) for all Nocardia species and 45.3% (58 of 128) for N. farcinica in a Spanish study using the ETEST stripe method (4), 5.4% (40 of 736) for all Nocardia and 4.0% (6 of 149) for N. farcinica in a French study using the disk diffusion method, and 9.3% (25 of 270) for all Nocardia species and 26.3% (10 of 38) for N. farcinica in Australia using broth microdilution methods (11). Discrepancies of resistance rates in different studies might be explained by several factors. First, unsatisfactory reproducibility of the ETEST stripe test or disk diffusion method, although user-friendly in the routine clinical laboratory, has been documented (22). Second, the lack of reproducibility of the broth microdilution method for Nocardia species has been also analyzed due to the inherent slow growth characteristics of the species and technical difficulties (23). Put together, AST is a helpful guide for nocardiosis treatment, but it should be interpreted with caution.

Although TMP-SMX is the usual nocardiosis treatment option, other antibiotics might be considered due to the occurrence of sulfonamide drug allergies and resistance. Amikacin has been used successfully in combination with other agents in patients with nocardiosis involving immunocompromised or aging hosts. It exhibits excellent *in vitro* activity against all species of Nocardia, with the exception of the N. transvalensis complex (N. wallacei in our study) and only 4 amikacin-resistant isolates in the current study, similar to that reported previously (1, 13, 24). Furthermore, linezolid is efficacious in the treatment of moderate to severe nocardiosis (25), often showing 100% drug susceptibility to Nocardia species in previous and current studies (6, 16). Consequently, linezolid and amikacin could be potentially used for empirical treatment of nocardiosis in China. Linezolid is sometimes used in severe nocardiosis alone or in combination with trimethoprim-sulfamethoxazole or other drugs. Amikacin is seldom used alone due to its relative lack of penetration into sites of infection (e.g., the central nervous system) and toxicities.

The β-lactam antibiotics were sometimes used as an alternative to TMP-SMX. However, imipenem showed different *in vitro* activity performance in different studies and against different Nocardia species (6, 11–13). N. brasiliensis is often resistant to imipenem with an “R” resistant pattern (15). The Nocardia species in our data revealed a resistance rate as high as 40.5% to imipenem. For imipenem, the poor performance of MIC repeatability was observed. Imipenem is known to be unstable in some liquid media, and this instability likely contributes to the high MICs seen in AST, especially the prolonged incubation length (over 3 to 4 days) sometimes required by some Nocardia isolates (26, 27). A previous study revealed there might be false resistance of N. cyriacigeorgica, N. farcinica, and N. wallacei isolates to imipenem (28). Therefore, in our study, the endpoint of imipenem should be read as early as the growth in the control plate meet the requirement. Furthermore, several drugs have been shown to give inconsistent results or false resistance with Nocardia spp. Ceftriaxone results have proven difficult to interpret consistently with N. cyriacigeorgica, N. brasiliensis and N. wallacei, which may result in reports of false resistance (28). Moreover, our isolates showed varied susceptibilities to other β-lactam antibiotics, including amoxicillin-clavulanic acid (40.3% resistance rate) and ceftriaxone (40.3% resistance rate), indicating that an AST should be conducted before usage.

As for fluoroquinolones, especially moxifloxacin, no drug pattern has been designated in CLSI standard M62 (15), and the resistance rate is often species specific, in line with previous studies (6, 11–13). In our study, moxifloxacin revealed a high susceptibility rate to N. farcinica (90.3%) and N. brasiliensis (94.4%) but a poor susceptibility rate to other Nocardia species.

Nocardia taxonomy has been linked to specific patterns of antimicrobial susceptibility patterns (13, 15, 29), as presented by Brown-Elliott et al. (1) and others (6, 11–13, 24, 29). We also noted a strong correlation between the drug pattern types and Nocardia species identification within our data. However, some discrepancies were also noted between species designation and susceptibility pattern; therefore, if possible, the AST should be performed for the best therapeutic option in nocardiosis. Taken together, the results indicate that species identification for Nocardia is often predictive of antimicrobial susceptibility. If the susceptibility testing results are contrary to the well-recognized patterns, they should be explained with caution and confirmed. The accurate identification of the infecting species and the determination of its susceptibility to antimicrobial agents, given a large number of strains with atypical patterns, are crucial if patients with nocardiosis are to be successfully treated. In addition, this study also presented the antimicrobial susceptibility for isolates of four unidentified Nocardia species. Collectively, these isolates represent approximately 1.0% of all Nocardia isolates.

Our study is limited by several factors: the lack of representation of all species within the genus Nocardia and the fact that the majority of the isolates were collected from a few provinces/cities in China. Correspondingly, the national surveillance program should be further developed.

In conclusion, the present study is, to date, the largest one, with a sample size of 441 nocardiosis strains covering 13 years and 21 provinces/cities in China. The looming threat posed by Nocardia isolates resistant to TMP-SMX and β-lactam antibiotics should be noted; nevertheless, the AST methods must be standardized. This study will help explain the diversity and antibiotic resistance profiles of Nocardia species distributed in China and aid decision-making in the context of empirical treatment.

## MATERIALS AND METHODS

### Strains collection and identification.

**FIG 3:**
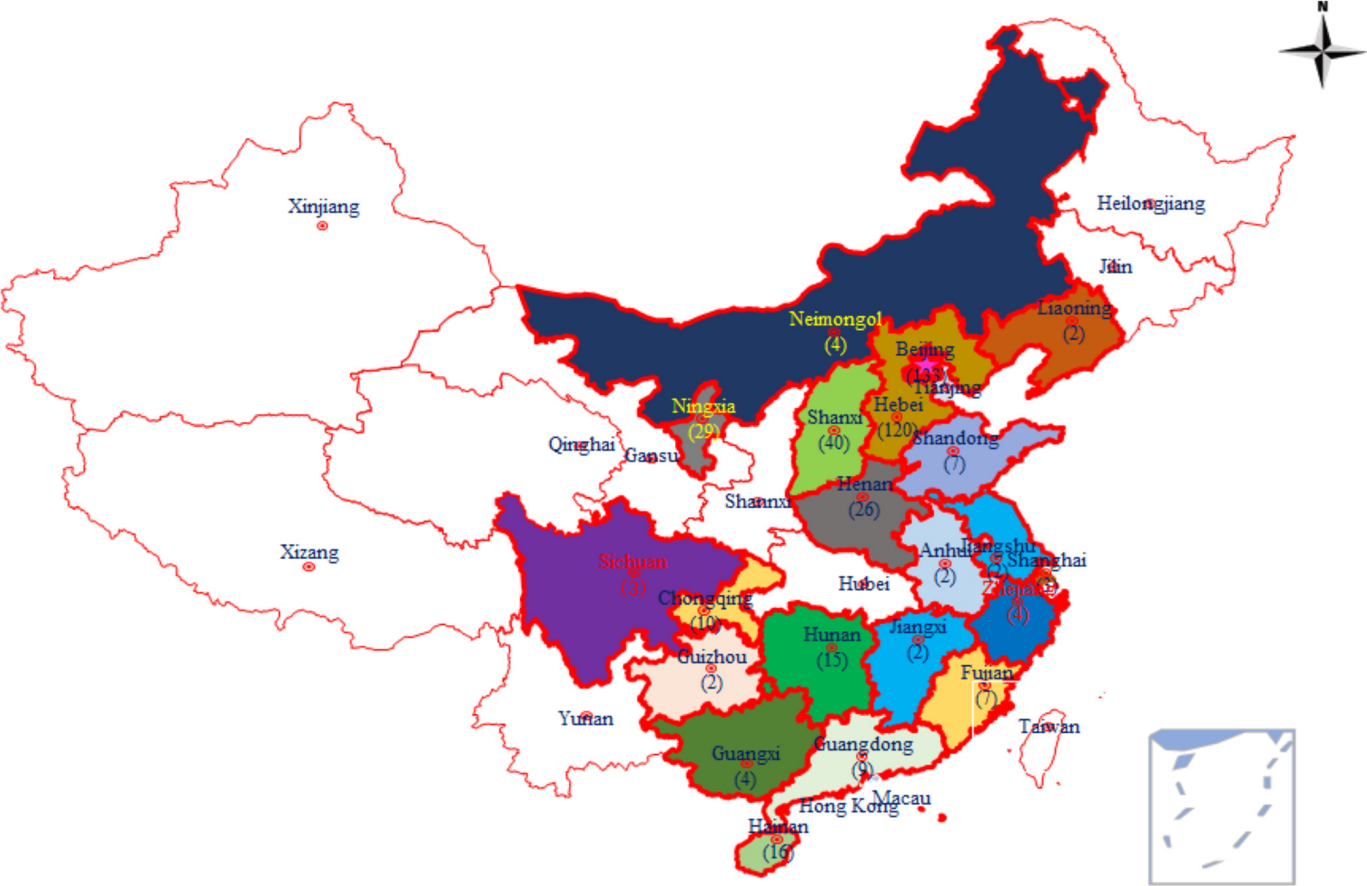
Geographical locations and distribution of *Nocardia* spp. from patients diagnosed with nocardiosis in 21 cities/provinces in China between 2009 and 2021. The color-highlighted cities/provinces represent those where *Nocardia* isolates were collected, with the number of strains provided in parentheses. The map in Fig. 3 was drawn by the authors and edited with Adobe Photoshop.

From September 2009 to March 2021, 441 nonrepetitive isolates of Nocardia were recovered from clinical samples from 21 provinces, autonomous regions, and municipalities of China, as shown in Fig. 3. All of the collected strains were sent for further study to the Laboratory of Clinical Microbiology and Infectious Diseases at China-Japan Friendship Hospital or the Department of Clinical Laboratory at the Second Hospital of Hebei Medical University.

### Primary identification by using matrix-assisted laser desorption ionization-time of flight mass spectrometry (MALDI-TOF MS).

The bacteria isolation and culture protocol were performed as described previously (2). Briefly, the collected isolates were inoculated into Columbia blood agar plates (Oxoid, UK) and cultured at 35°C in an incubator for 48 h, or longer if necessary. The fresh colonies were collected and identified based on both colony morphology and MALDI-TOF MS (Bruker Daltonik, Bremen, Germany) following the manufacturer’s suggested recommendations. The ethanol/formic acid extraction method was applied as described previously (30). If MALDI-TOF MS failed to identify the Nocardia strains to the species, the targeted sequencing was applied for identification.

### DNA extraction and species identification by sequencing.

Genomic DNA was extracted from each unidentified Nocardia strain following the protocol described previously with some modifications (30, 31). Definitive identification was performed by sequencing the full length of the 16S rRNA gene, if necessary, complemented with sequencing of the subunit A of SecA preprotein translocase (*secA1*) gene, with the primer sequences and PCR conditions described previously (2, 32, 33). The PCR products were purified, and sequencing was performed by using ABI 3730 DNA analyzer. The sequences were compared using the BLAST algorithm with the database in the NCBI GenBank (http://www.ncbi.nlm.nih.gov). Species identification was based on the similarity value of equal to or greater than 99.6% for 16S rRNA and 99.0% for *secA1* (33, 34). The taxonomy for Nocardia species was defined in line with a recent review by Conville et al. (7). If the above criteria of identification could not be met, the Nocardia strains will be defined as unidentified.

### Analysis of whole-genome sequencing (WGS).

WGS of four unidentified Nocardia sp. isolates was performed on a HiSeq sequencer (Illumina) following the manufacturer’s instructions. FASTQ format files of each sample were independently assembled using a *de novo* SPAdes genome assembler (version 3.13.1).

### Antibiotic susceptibility testing (AST).

MICs were determined using the commercial Sensititre Rapmyco microdilution panel (Thermo Fisher, Inc., Cleveland, OH) following the incubation conditions according to the manufacturer’s instructions. Briefly, the fresh colonies of the Nocardia strains were transferred to sterile 0.9% sodium chloride water and then subjected to repeated vortexing until there were no visible flakes, particles, or deposits. Vortexing with glass beads help to gain a homogeneous suspension. If large clumps remain after vortexing vigorously, they should be allowed to settle, and the supernatant employed for the inoculum suspension.

Afterward, the supernatant was adjusted to the turbidity of 0.5 McFarland Standard. The tested drugs included TMP-SMX, linezolid, ciprofloxacin, imipenem, moxifloxacin, cefepime, cefoxitin, amoxicillin-clavulanic acid, amikacin, ceftriaxone, doxycycline, minocycline, tigecycline, tobramycin, and clarithromycin. Nocardia asteroides ATCC 19247), Staphylococcus aureus ATCC 29213, and Escherichia coli ATCC 35218 (for amoxicillin-clavulanate) were used as quality control. The AST was conducted under 35°C in ambient air for 2 to 3 days, or more if necessary. While moderate growth was observed, appearing as turbidity or a deposit of cells at the bottom of the well, two well-trained microbiologists read plates (23, 28). The MIC of TMP-SMX was read at approximately 80% growth inhibition. The results were interpreted according to the Clinical and Laboratory Standards Institute (CLSI) interpretive criteria for Nocardia (CLSI standard M62, 2018 [15]). When the MIC results change in interpretation (e.g., susceptible, intermediate, and resistant) from the CLSI expected antimicrobial susceptibility patterns for that species in CLSI standard M62, testing should be repeated (15).

Furthermore, the susceptibility patterns of Nocardia spp. in our study were compared with those described in previous studies (6, 11–13). The enrolled criteria included the studies in which the broth microdilution method was employed with commercial Sensititre Rapmyco microdilution panel from Thermo Fisher. The sample size was >100, and the detailed susceptibility rate of antibiotics could be extracted.

### Statistical analysis.

The MIC data of each antibiotic were recorded and analyzed by WHONET 5.6 software, and MIC_50_ and MIC_90_ were also calculated, defined as the MICs of a given agent that inhibits the growth of 50% and 90% of the isolates, respectively. Furthermore, the distribution of Nocardia species, as well as the ages and infection types, were illustrated by using GraphPad Prism version 8.01.

### Data availability.

The original contributions presented in the study are all included in the article. Further inquiries can be directed to the corresponding author.
